# Comparison of Perinatal Outcome of Preterm Births Starting in Primary Care versus Secondary Care in Netherlands: A Retrospective Analysis of Nationwide Collected Data

**DOI:** 10.1155/2014/423575

**Published:** 2014-12-30

**Authors:** A. J. van der Ven, J. M. Schaaf, M. A. van Os, C. J. M. de Groot, M. C. Haak, E. Pajkrt, B. W. J. Mol

**Affiliations:** ^1^Department of Obstetrics and Gynaecology, Academic Medical Center, P.O. Box 22700, 1100 DE Amsterdam, Netherlands; ^2^Department of Medical Informatics, Academic Medical Center, Amsterdam, Netherlands; ^3^Department of Obstetrics and Gynaecology, VU University Medical Center, Amsterdam, Netherlands; ^4^Department of Obstetrics and Gynaecology, Leiden University Medical Center, Leiden, Netherlands; ^5^School of Paediatrics and Reproductive Health, University of Adelaide, SA 5000, Australia

## Abstract

*Introduction*. In Netherlands, the obstetric care system is divided into primary and secondary care by risk level of the pregnancy. We assessed the incidence of preterm birth according to level of care and the association between level of care at time of labor onset and delivery and adverse perinatal outcome. *Methods*. Singleton pregnancies recorded in Netherlands Perinatal Registry between 1999 and 2007, with spontaneous birth between 25^+0^ and 36^+6^ weeks, were included. Three groups were compared: (1) labor onset and delivery in primary care; (2) labor onset in primary care and delivery in secondary care; (3) labor onset and delivery in secondary care. Multivariable logistic regression analyses were performed to calculate the risk of perinatal mortality and Apgar score ≤4. *Results*. Of all preterm deliveries, 42% had labor onset and 7.9% had also delivery in primary care. Women with labor onset between 34^+0^ and 36^+6^ weeks who were referred before delivery to secondary care had the lowest risk of perinatal mortality (aOR 0.49 (0.30–0.79)). Risk of perinatal mortality (aOR 1.65; 95% CI 1.20–2.27) and low Apgar score (aOR 1.95; 95% CI 1.53–2.48) were significantly increased in preterm home delivery. *Conclusion*. Referral before delivery is associated with improved perinatal outcome in the occurrence of preterm labor onset in primary care.

## 1. Introduction

Spontaneous preterm birth (PTB), defined by the World Health Organization as birth before 37 completed weeks of gestation, is one of the main causes of perinatal death in the developed countries [1]. PTB has multifactorial causes and a heterogeneous outcome [2–7]. For surviving preterm neonates, there may be significant health consequences with lasting disabilities, including respiratory problems, hearing and vision impairment, cerebral palsy, and mental retardation [2, 3].

In 2008, the EURO-PERISTAT study showed that perinatal mortality in Netherlands was relatively high when compared to other European countries [8]. The pathways leading to this are not completely clear and under investigation [9–11].

It has been suggested that PTB (<28 weeks) is a major cause of the low ranking of Netherlands in the EURO-PERISTAT study [12], although the incidence of PTB in Netherlands is comparable with the rest of Europe [13]. Among singleton pregnancies, the incidence of preterm deliveries is 6.0% and of very preterm deliveries, that is, before 32 completed weeks, 0.9% [10].

The Dutch obstetric care system is different from most other developed countries, since the level of care is organised according to the presence or absence of risk factors in medical and/or obstetrical history. However, it is not known if levels of care at labor onset and at time of delivery are determining factors in the pathway to adverse perinatal outcome.

The Dutch obstetric care system is structured as follows. Pregnant women without risk factors are under surveillance by primary obstetric care providers (midwives and in rural areas a few general practitioners who provide primary obstetric care (GPs)). Women with an a priori high risk profile, due to their medical or obstetrical history, receive secondary care from the beginning of pregnancy. If complications occur during pregnancy, women will be referred to secondary care during pregnancy. As risk factors can arise at any time, risk-selection remains a continuous process during pregnancy and delivery. Indications for primary and secondary care have been formulated by consensus between primary and secondary care providers in the so-called “List of Obstetric Indications” (LOI) [14] and all professionals involved in pregnancy care are bound to follow these guidelines. In the LOI, the policy regarding PTB is set as follows.

- Preterm labor defined as preterm rupture of membranes and/or preterm contractions before 37 completed weeks of gestation is of its nature an indication for referral to secondary care.
- Women with previous PTB before 33 completed weeks have an indication for secondary care from the beginning of prenatal care until 37 weeks.
- Women with previous PTB after 33 completed weeks can be cared for in primary care.

Women attending primary care will visit their midwife or GP on a regular consulting basis at the practice premises, which is in most cases within a twenty-minute drive of their home. In case of predefined changes in their normal pregnancy process, they will contact their primary care giver. Subsequently, the pregnant woman will be invited for an extra check or the care provider will visit her at home to assess the changes in order to distinguish between normal and abnormal changes and to assess if referral to secondary care is mandatory. This takes time and may sometimes be the cause of delay when referral to secondary care is indicated because of impending preterm delivery. In case of precipitous PTB, the midwife or GP will evaluate whether there is sufficient time for transport to hospital or if not will accept PTB at home. Thus in case of rapidly progressing spontaneous PTB there is always a risk of an unintended home delivery.

In contrast, patients in secondary care will contact their attending obstetricians by phone in case of signs of imminent PTB. After triage, they will be advised to come straight to hospital, thereby reducing delay and consequently reducing the risk of an unexpected home delivery. However, precipitous preterm labor may also occur in secondary care patients. In case of insufficient time to reach the hospital, the obstetrician will request the nearest midwife on call to assist the pregnant woman with her delivery at home. Unfortunately, unintended home delivery is not registered as such in Netherlands Perinatal Registry.

To gain insight in the incidence of PTB and if levels of care at labor onset and at time of delivery are determining factors in the pathway to adverse perinatal outcome, we aimed to conduct an exploratory study.

## 2. Methods

### 2.1. Dataset

This study was performed in a nationwide retrospective cohort using Netherlands Perinatal Registry (PRN). The PRN consists of population-based data containing information on pregnancies, deliveries, and (re)admissions until 28 days after birth. The PRN database is obtained by a validated linkage of 3 different registries: the midwifery registry (LVR1), the obstetrics registry (LVR2), and the neonatology registry (LNR) of hospital admissions of newborns [15]. The coverage of the PRN is about 96% of all deliveries in Netherlands. All data contained in PRN are voluntarily recorded by the caregiver during prenatal care, delivery, and perinatal period. The data are sent annually to the national registry office, where a number of range and consistency checks are conducted [16].

### 2.2. Inclusion and Exclusion Criteria

For this study, all singleton spontaneous PTBs between 1 January, 1999, and 31 December, 2007, were selected. PTB was defined as birth before 37 completed weeks of gestation (before 259 days). Spontaneous onset of birth was considered in case of spontaneous contractions or spontaneous rupture of membranes. Women with iatrogenic PTB as a consequence of induction of labor or an elective Caesarean section were not included. Gestational age data were predominantly based on the date of the last menstrual period and/or the crown-rump length (CRL). We excluded all pregnancies of women who delivered before gestational age (GA) of 25^+0^ as this was the threshold for fully active perinatal treatment during the study period.

In this study, we focused solely on spontaneous PTB (with or without pPROM). Pregnancies with an unknown gestational age or resulting in antenatal intrauterine fetal death or the birth of a child weighing less than 500 grams were excluded from this study. Antenatal intrauterine fetal death is registered as such in the PRN. In the calculation of perinatal mortality, all fetuses with a positive heart rate at the start of the delivery, confirmed by auscultation or any sign of life observed after birth, were included.

Moreover, we excluded all fetuses with congenital abnormalities as well as all cases with an unknown obstetric care provider.

### 2.3. Definition of Determinant

The primary variable of interest was the level of care in which labor and/or delivery took place. We defined three categories: (1) onset of labor and delivery in primary care, (2) onset of labor in primary care and delivery in secondary care (intrapartum referral), and (3) onset of labor and delivery in secondary care.

### 2.4. Definition of Outcome Measures

The primary outcome measure was perinatal mortality, defined as intrapartum or neonatal mortality in the first week of life. The secondary outcome measure was an Apgar score less than or equal to 4 after 5 minutes as criterion for diagnosing perinatal asphyxia.

### 2.5. Statistics

Baseline characteristics of the three patient groups under investigation were assessed. We analysed maternal age, parity, maternal ethnicity (European white versus other), socioeconomic status, and living in a deprived neighbourhood (yes or no, based on four-digit zip codes and a public list of deprived neighbourhoods issued by the Dutch government) according to both outcome measures. To analyse the association between variables and spontaneous PTB related perinatal mortality and low Apgar score, we performed univariable logistic regression analysis. Subsequently, multivariable regression analysis was performed to obtain adjusted odds ratios (aOR). We calculated the incidence of our outcome measures for each individual year to investigate trends over time. Data were analyzed using SAS statistical software package version 9.2 (SAS Institute Inc., NC, USA).

## 3. Results

Between 1999 and 2007, there were 52,397 births that met our inclusion criteria (Table 1).

Table 2 shows the baseline characteristics of the study population. In total, 22,121 (42%) of all spontaneous singleton PTBs had labor onset in primary care.

**Figure 1:**
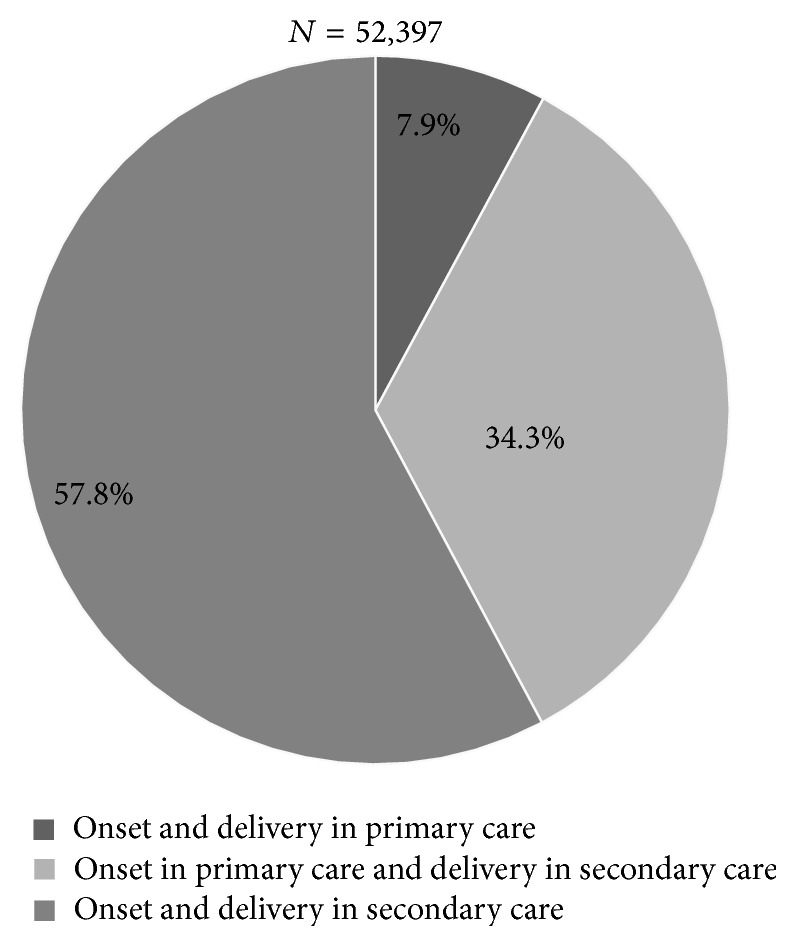
Study population (PTB 25^+0^–36^+6^) divided by level of care at onset and delivery.

Of those, 4,134 (7.9%) were subsequently also delivered in primary care (Figure 1).

The proportion of perinatal death and Apgar score less than or equal to 4 are also presented in Table 2.

There were no major differences in baseline characteristics between cases with onset in primary care and those with onset in secondary care, making the two groups comparable at the onset of delivery. The median gestational age at delivery was 36^+2^ weeks in the group with onset and delivery in primary care versus, respectively, 35^+6^ in the group with onset in primary care and delivery in secondary care and 35^+3^ in the group with onset and delivery in secondary care (*P* value < 0.0001). Of all PTBs, 20.9% (10,957/52,397) occurred before a GA of 34^0^ weeks.

Table 3 shows the rates and odds ratios for Apgar score less than or equal to 4 and perinatal mortality after adjustment for maternal age, parity, ethnicity, socioeconomic status, fetal sex, and gestational age.

After adjustment, nulliparous women had the lowest risk of perinatal mortality and Apgar score less than or equal to 4. Both risk of perinatal mortality and risk of low Apgar score were decreased if women were referred before delivery to a secondary care setting. However, the risk was significantly increased if onset of labor and delivery took place in primary care.

A subgroup analysis was performed for late PTBs, that is, between 34^+0^ and 36^+6^ weeks of gestation.  Table 4 shows the baseline characteristics of this group.

**Figure 2:**
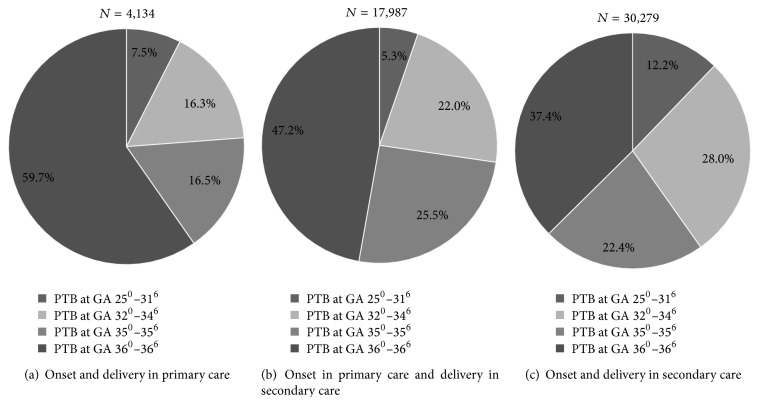
All preterm births (*N* = 52, 397) per level of care divided by gestational age (GA).

Of the total group of PTBs with onset in primary care, 86% (19,001/22,121) were late preterm compared to 74% (22,439/30,272) being late preterm with onset in secondary care. Within the group of PTBs with onset and delivery in primary care, almost 60% took place between GA of 36^+0^ and 36^+6^ weeks (Figure 2(a)).

Table 5 shows the adjusted odds ratios in the subgroup of late PTBs (GA 34^+0^–36^+6^).

Women with preterm onset of labor between 34^+0^ and 36^+6^ weeks, who were referred before delivery to secondary care, had the lowest risk of low Apgar score (aOR 0.72 (0.53–0.98)) and perinatal mortality (aOR 0.49 (0.30–0.79)). The risk for a 5-minute Apgar score less than or equal to 4 was significantly increased for women with onset and delivery in primary care comparing to onset and delivery in secondary care. The increased risk on perinatal mortality for women with onset and delivery in primary care reached the border of significance (aOR 1.61 (0.96–2.21)). In the subgroup of late PTBs, multiparous women (≥2) had the highest risk on perinatal mortality (aOR 2.14 (1.31–3.51)).

### 3.1. Trends

**Figure 3:**
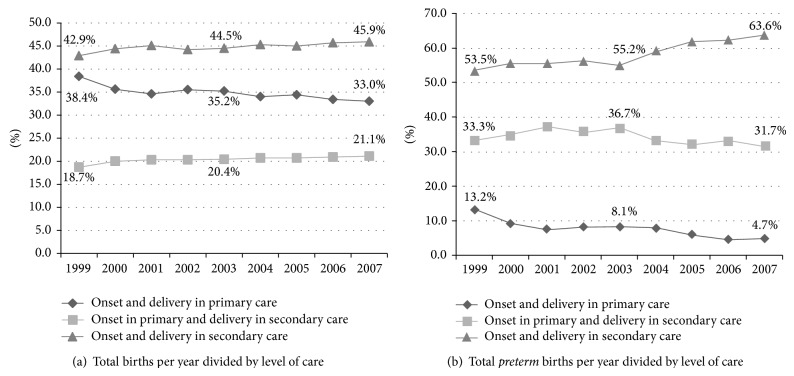
Trends over 1999–2007.

Figure 3(a) shows the trends over the years 1999–2007 in incidence of the total of all births according to the type of supervision at the time of onset of labor and delivery.  Figure 3(b) shows the trends in incidence of preterm births according to level of care.

The incidence of PTBs in a primary care setting has steadily decreased. In 1999, 38.4% of all deliveries took place under supervision of a midwife or GP; in 2007, this rate declined to 33%, indicating a relative decrease of 14%. However, of all PTBs in 1999, 13,2% had onset and delivery in primary care compared to 4.7% in 2007, a relative decrease of 64%. Although the overall share of primary care in the birthrate has declined over the years, the reduction in PTBs is stronger. Subsequently, the number of PTBs in a secondary care setting increased from 53.5% to 63.6% (a relative increase of 19%) while referrals from primary to secondary care during preterm labor showed a relative decrease of 5%.

## 4. Discussion

### 4.1. Principal Findings

Our study shows that the risk of adverse perinatal outcome after spontaneous PTB was lowest for women with labor onset in primary care who were referred to a secondary care setting before delivery. The risk of perinatal mortality and the risk of low Apgar score after 5 minutes were significantly increased for those women with both labor onset and delivery in primary care compared to women with labor onset and delivery in a secondary care setting.

Of all spontaneous singleton PTBs, 42.2% (Figure 1(a)) had labor onset in primary care and 7.9% of these births subsequently ended in primary care. Of all PTBs in primary care, 85% were late preterm (34^+0^–36^+6^ weeks) and almost 60% between 36^+0^ and 36 ^+6^ weeks (Figure 2(a)).

### 4.2. Strengths and Weaknesses

Our study was based on data of a large population-based national perinatal registry. Most professionals in obstetrical care contribute to the PRN and it thus comprises approximately 96% of all pregnancy and birth characteristics in Netherlands. The 4% of missing birth data are due to 1-2% nonreporting general practitioners and 2-3% nonreporting midwives.

The Dutch obstetrical system has received a great deal of attention over the last few years, both nationally and internationally, mainly because of its relatively high perinatal mortality compared to other developed countries and the possible contribution of our obstetric care system with distinct differentiation between primary and secondary care. As far as we know, this is the first study that examines the incidence of PTB according to level of care in which the share of both levels in perinatal mortality and low Apgar score is studied.

The method of pregnancy dating can influence the incidence of PTB [17–19]. In the last decade, the vast majority of women in Netherlands received an early pregnancy ultrasound to confirm or change the gestational age that was based on date of last menstrual period. Since this strategy did not change during the study period, the effect of miscalculated gestational age on the studied outcome of PTB should be marginal.

During the study period, approximately 83% [20] of all pregnant women received primary care at the onset of pregnancy. We assume that the majority of the remaining 17% of women had a high risk profile according to the LOI guidelines (e.g., diabetes, hypertension, multiple pregnancies, previous Caesarean section, and chronic disease) at the onset of the pregnancy, including a history of PTB before 33 completed weeks. During pregnancy, changing level of care is not uncommon and can go both ways, but overall this generally results in more referrals from primary to secondary care than vice versa. Consequently, at the onset of labor, 39.5% of all pregnant women are under obstetrician-led care. Some of those women may have risk factors related to a higher risk of (indicated) preterm labor or risk factors that may affect neonatal outcome. In our study, we evaluated the difference in perinatal outcome in spontaneous PTB according to the level of care at the onset of delivery and at delivery. We excluded induced PTBs in this study but did not adjust for risk factors in medical or obstetrical history nor for pregnancy related complications and this may have influenced our neonatal data. If we compare our three groups, it is evident that (initially low risk) pregnancies with onset and preterm delivery in primary care had the worst outcome. In the crude analysis, the group with onset in primary care and preterm delivery in secondary care had the best outcome. This concerns all low risk pregnancies with a referral to secondary care before delivery, in case of spontaneous PTB. However, after adjusting (Tables 3 and 4), there was no significant difference between this group and the group with onset and delivery in secondary care. This is remarkable because the latest group concerns women who are at increased risk as a result of their medical or obstetric history. If we had adjusted for risk factors, the results would probably be even more in favour of the group with onset and delivery in secondary care. Comparing neonatal outcome based on setting at onset of delivery implies comparing outcome in low and high risk patients, without correction for risk factors. However, we do not feel this would undermine our results; namely, healthy low risk women with PTB cared for in primary care do not have better perinatal outcome than women at increased risk as a result of a number of unspecified conditions cared for in secondary care. On the contrary, the adjusted risk outcomes are worse if PTB occurs in primary care setting although referral before delivery affects the results positively.

We suggested that precipitous PTB could be one of the reasons why delivery ended at home by midwifery-led care. It may take some time for the midwife to travel to the pregnant woman for further examination after the first call and it is possible that there is not enough time for hospital referral. Nonetheless, a precipitous (preterm) birth can also occur in secondary care. If the obstetrician suspects a precipitous (preterm) birth at the first call while the patient is still at home, there are two options: firstly, to send an ambulance for emergency transport to the hospital and secondly, to alert the nearest midwife with the request to assist the woman at home. In that case, the birth is registered as a primary care birth. The probability that there is no medical assistance available at the time of birth is very rare but occurs incidentally. Unfortunately this is not registered consequently.

### 4.3. Relation to Other Studies

In our study, we found lower odds of perinatal mortality in case of PTB for ethnicity other than European white and the odds of a 5-minute Apgar score less than or equal to 4 reached the border of significance. This is entirely consistent with the study of Schaaf et al. [21]. They concluded that although African and South Asian women had an increased risk of PTB, they had a decreased risk of adverse neonatal outcome after PTB. Mediterranean women had a decreased risk of PTB when compared to European white women but also a significant decreased risk of subsequent adverse neonatal outcome.

Several studies have compared the outcome associated with planned home births and planned hospital births in low risk women [22, 23]. Planned home birth was associated with less medical interventions, but the interpretation of perinatal outcome differed in these studies [24, 25]. Evers et al. [26] have also investigated the incidence of perinatal mortality and morbidity in low risk term pregnancies supervised by a midwife and compared those to high risk pregnancies in secondary care. Their study suggested that the Dutch system of risk selection, which is based on two independent and separately working obstetric care levels, is not as effective as it should be. However, as the authors themselves stated in a reply on comments on their study, a causal association between the results and the obstetric care system cannot be shown because of limitations of the chosen study design. The authors indicated that little is known regarding delay in primary as well as in secondary care after referral and/or during transport. Also, essential information could be lost during referral causing inadequate treatment in the hospital. It is not known to what extent these factors have contributed to adverse outcomes.

de Jonge et al. [14] compared perinatal mortality and morbidity between planned home and hospital births in low risk pregnancies in Netherlands. This study did not show an increased risk for adverse outcome in planned home birth in the Dutch obstetric care system with well-trained midwives and good referral and transportation system.

Comparability of our results with the results of these studies and others [22–25] on perinatal outcome according to the intended place of birth is limited because PTB in primary care is not planned as such. Preterm onset of labor is an indication for referral to a secondary care setting because of the risks of PTB for the neonate. None of the studies compared the outcome of PTB under primary care versus secondary care. However, our study does show that the Dutch obstetric care system is well organized, since the group of low risk women with onset in primary care and PTB in secondary care had the best neonatal outcome as expected.

The number of women with onset of labor in primary care decreased from 60.5% in 1999 to 54.3% in 2007 [20], a relative decrease of 10.3%. Meanwhile, the incidence of spontaneous singleton PTB between 25^+0^ and 37^+0^ weeks with onset and delivery in primary care decreased during the study period from 13.2% to 4.7%, resulting in an absolute decrease of 7.5% and a relative decrease of 64%. These data indicate that referral by primary care providers in case of (imminent) PTB has markedly improved.

The epidemiology of PTBs and trends in the last decade are well investigated [4–7, 9]. In Netherlands, the risk on adverse outcome in preterm singleton pregnancies decreased significantly during the study period from 8.0% to 7.7% [8]. The reduction was mainly due to a decrease of two types of PTB: first of all the decrease of spontaneous PTB with or without preterm prelabor rupture of membranes (pPROM) from 3.6% to 3.1% and secondly a decrease in PTB within gestational age of 34–36 weeks from 4.6% to 4.2%.

During our study period, there was a relative decrease of PTBs with onset and delivery in primary care of 64% (13.2% of all PTBs in 1999 versus 4.7% in 2007). Both abovementioned trends are partly responsible for this decrease because PTBs in primary care setting are always spontaneous PTBs and 85% of them occur between 34^+0^ and 36^+6^ weeks. Another contributor is the overall decreasing share of primary care in birth assistance. However, all these factors do not explain the 64% relative decrease as we mentioned before. We can only welcome the decline, given the poorer outcome of PTBs in primary care setting.

Even so, the Dutch perinatal mortality declined steadily over the years 2000–2006, according to the study of Ravelli et al. [10]. These dates indicate that the decrease in perinatal mortality risk was most prominent among births with congenital anomalies (45% decline), among term births (25% decline) and among PTBs with 32.0–36.6 weeks' gestation (30% decline).

This positively influenced the primary outcome of our study.

### 4.4. Meaning of the Results and Proposal for Future Research

Our study shows that the risk of perinatal mortality and Apgar score less than or equal to 4 after spontaneous PTB is higher if birth takes place in primary care. In late PTB, the risk of low Apgar score is still higher than for births in a secondary care setting. Because of its impact on perinatal outcome and costs, reducing PTB is a worldwide challenge in obstetrics and a topic of research [27–30].

One of the most important aspects of midwifery care in Netherlands is risk selection. Midwives in Netherlands are independent practitioners, qualified to provide full maternity care to all women whose pregnancy and childbirth are uncomplicated. When risk factors occur in pregnancy or during birth, women are referred to a secondary care setting.

In case of imminent PTB, referral to an obstetrician is indicated according to the Dutch guidelines. Nevertheless, there are factors that may hamper referral before delivery to a hospital for delivery.

Firstly, patient related factors: we hypothesize that pregnant women may be insufficiently aware of the signs associated with the onset of labor and when to contact the obstetric caregiver or did not understand the advice of her obstetric caregiver, maybe due to a language barrier. In these cases, it is useful to examine in which way pregnant women, especially nulliparous, could be informed to raise the awareness.

Secondly, obstetric care related factors: the obstetric caregiver did not think of imminent PTB with the specified complaints or did not respond adequately enough or underestimated the problem and was subsequently confronted with a preterm parturition. In case of late PTB, there might be acceptation of the parturition by the obstetric caregiver, but it is not known to what extent this occurs.

Thirdly, unforeseeable circumstances: the fastness of the delivery left no time for referral to the hospital and transportation of the woman in labor was not a safe option anymore.

Ravelli et al. [31] investigated the impact of travel time, from home to hospital during delivery, on neonatal outcome in Netherlands. They concluded that there is a significant association between a travel time of 20 minutes or longer and adverse outcomes.

Considering all conceivable reasons for preterm delivery in a primary care setting, it is of major importance that both midwives and pregnant women are aware of the risk outcome of (late) spontaneous PTB.

A risk assessment to predict PTB in low risk women without any symptoms would be very valuable but a good risk selection tool is not available yet. Despite all research, the clinical applicability of the previously developed prediction models is still limited [27, 32, 33]. Therefore, the focus should be on increasing awareness in patients and midwives of potential signals of impending PTB and of the risks of (late) PTB in primary care setting.

## 5. Conclusion

Our study shows that the risk of adverse perinatal outcome was significantly increased after spontaneous preterm birth for healthy women with low risk singleton pregnancies with labor onset and delivery in primary care. For women with labor onset in primary care who were referred to a secondary care setting before delivery, the adjusted risk of adverse perinatal outcome is comparable to those with onset and delivery in secondary care setting. We recommend improving the awareness of both the complaints and, above all, the risks associated with preterm labor of primary obstetric care providers as well as of the pregnant women in order to achieve an increasing referral before delivery to secondary care, because this improves perinatal outcome after preterm onset of labor.

## Figures and Tables

**Table 1:** Study population.

Total births in Netherlands 1999–2007			**1,633,636**	
Included births at gestational age of 25^+0^–36^+6^			**123,388**	7.6%

Exclusion of the total births between 25^+0^ and 36^+6^ wks	*N*	%		

Multiple pregnancies	30,041	24.4%		
Congenital anomalies	6,367	6.8%		
Antenatal death	3,400	4%		
Induction of labor and primary Caesarean section	31,039	37%		
Unknown level of care at onset of labor and delivery	144	0%		
	70,991		**70,991**	

Study population			**52,397**	3.2%

**Table 2:** Baseline characteristics and outcome of 52,397 women with preterm delivery (GA 25^+0^–36^+6^).

Population 1999–2007	Total	Onset and delivery	Onset in prim. care	Onset and delivery
	*N*/%	Primary care	Delivery in sec. care	Secondary care
Total GA 25^0^–36^6^	52397 (100)	4134 (7.9)	17987 (34.3)	30279 (57.8)
minus exclusions *n* (%)				
Maternal age				
<25 years, *n* (%)	7573 (14.5)	603 (14.6)	2858 (15.9)	4112 (13.6)
25–29 years, *n* (%)	16662 (31.8)	1311 (31.7)	6499 (36.1)	8852 (29.2)
30–34 years, *n* (%)	19432 (37.1)	1568 (37.9)	6521 (36.3)	11343 (37.5)
≥35 years, *n* (%)	8730 (16.7)	652 (15.8)	2109 (11.7)	5969 (19.7)
Parity				
0, *n* (%)	31115 (59.4)	2365 (57.2)	12386 (68.9)	16364 (54.1)
1, *n* (%)	14407 (27.5)	1193 (28.9)	4047 (22.5)	9167 (30.3)
2+, *n* (%)	6875 (13.1)	576 (13.9)	1554 (8.6)	4745 (15.7)
Ethnicity				
European white, *n* (%)	43898 (83.8)	3377 (81.7)	15381 (85.5)	25140 (83.0)
Socioeconomic status				
High, *n* (%)	11976 (22.9)	929 (22.5)	4119 (22.9)	6928 (22.9)
Medium, *n* (%)	26080 (49.8)	2099 (50.8)	9184 (51.0)	14797 (48.9)
Low, *n* (%)	14341 (27.4)	1106 (26.8)	4784 (26.0)	8551 (28.2)
Deprived neighborhood				
Yes, *n* (%)	3730 (7.1)	276 (6.7)	1129 (6.3)	2325 (7.9)
Fetal sex				
Male fetal sex, *n* (%)	29686 (56.7)	2337 (56.5)	10295 (57.2)	17054 (56.7)
Apgar score ≤4 after 5 min				
Apgar ≤4, *n* (%)	805 (1.5)	94 (2.3)	158 (0.9)	553 (1.8)
Perinatal death				
Perinatal death, *n* (%)	575 (1.10)	58 (1.4)	99 (0.6)	418 (1.4)

**Table 3:** Unadjusted and adjusted^*^ odds ratios (OR) for 5-minute Apgar score ≤4 and perinatal mortality (GA 25^+0^–36^+6^).

Characteristics	Apgar score ≤4 after 5 minutes			Mortality intrapartum and ≤7 dgn postpartum		
GA 25^+0^–36^+6^ *N* 52397	*N* (%)	Unadjusted OR (95% CI)	Adjusted OR (95% CI)	*N* (%)	Unadjusted OR (95% CI)	Adjusted OR (95% CI)
Parturition	805 (1.54)			575 (1.1)		
Onset and delivery in primary care	94 (0.18)	1.25 (1.00–1.56)	1.95 (1.53–2.48)	58 (0.11)	1.02 (0.77–1.34)	1.65 (1.20–2.27)
Onset primary and delivery in sec. care	158 (0.3)	0.48 (0.40–0.57)	0.89 (0.74–1.08)	99 (0.19)	0.40 (0.32–0.49)	0.86 (0.67–1.09)
Onset and delivery in secondary care	553 (1.06)	Reference	Reference	418 (0.8)	Reference	Reference
Maternal age						
<25 years		1.38 (1.11–1.70)	1.11 (0.88–1.40)		1.44 (1.12–1.86)	1.14 (0.85–1.52)
25–29 years		Reference	Reference		Reference	Reference
30–34 years		1.08 (0.90–1.29)	1.02 (0.85–1.27)		1.13 (0.92–1.40)	1.06 (0.84–1.34)
≥35 years		1.40 (1.14–1.72)	1.10 (0.88–1.38)		1.50 (1.18–1.91)	1.08 (0.81–1.42)
Parity						
0		0.78 (0.66–0.92)	0.74 (0.62–0.88)		0.80 (0.66–0.97)	0.74 (0.60–0.93)
1		Reference	Reference		Reference	Reference
2+		1.46 (1.2–1.8)	1.19 (0.95–1.48)		1.46 (1.2–1.8)	1.43 (1.10–1.87)
Ethnicity						
European white		Reference	Reference		Reference	Reference
Non-European white		1.36 (1.14–1.61)	0.88 (0.72–1.07)		1.27 (1.03–1.56)	0.69 (0.53–0.89)
Socioeconomic status						
High		Reference	Reference		Reference	Reference
Medium		1.11 (0.92–1.33)	1.07 (0.88–1.38)		1.04 (0.84–1.28)	0.98 (0.77–1.24)
Low		1.29 (1.05–1.57)	1.09 (0.87–1.35)		1.15 (0.91–1.46)	0.95 (0.73–1.25)
Fetal sex						
Female		Reference	Reference		Reference	Reference
Male		0.94 (0.81–1.08)	0.89 (0.77–1.04)		0.95 (0.81–1.12)	

^*^Adjusted for onset and location of delivery, maternal age, parity before study delivery, ethnicity, socioeconomic status, fetal sex, and gestational age.

**Table 4:** Baseline characteristics and outcome of 41,440 women with late preterm delivery (GA 34^+0^–36^+6^).

Population 1999–2007	Total	Onset and delivery	Onset in prim. care	Onset and delivery
	*N*/%	Primary care	Delivery in sec. care	Secondary care
Total GA 34^0^–36^6^	41440 (100)	3494 (8.4)	15507 (37.4)	22439 (54.2)
minus exclusions *n* (%)				
Maternal age				
<25 years, *n* (%)	5799 (14.0)	484 (13.9)	2393 (15.4)	2922 (13.0)
25–29 years, *n* (%)	13278 (32.0)	1117 (32.0)	5629 (36.3)	6532 (32.0)
30–34 years, *n* (%)	15496 (37.4)	1332 (38.1)	5654 (36.5)	8510 (37.9)
≥35 years, *n* (%)	6867 (16.6)	561 (16.1)	1831 (11.8)	4475 (19.9)
Parity				
0, *n* (%)	24453 (59.0)	1936 (55.4)	10527 (67.9)	11990 (53.4)
1, *n* (%)	11651 (28.1)	1065 (30.5)	3595 (23.2)	6991 (31.3)
2+, *n* (%)	5336 (12.9)	493 (14.1)	1385 (8.9)	3458 (15.4)
Ethnicity				
European white, *n* (%)	34881 (84.2)	2871 (82.2)	13252 (85.5)	18758 (83.6)
Socioeconomic status				
High, *n* (%)	9572 (23.1)	784 (22.4)	3575 (23.05)	5213 (23.2)
Medium, *n* (%)	20736 (50.0)	1789 (51.2)	7956 (51.3)	10991 (49.0)
Low, *n* (%)	11132 (26.9)	921 (26.4)	3976 (25.6)	6235 (27.8)
Deprived neighborhood				
Yes, *n* (%)	2853 (6.9)	225 (6.4)	968 (6.2)	1660 (7.4)
Fetal sex				
Male fetal sex, *n* (%)	23143 (55.9)	1965 (56.3)	8807 (56.8)	12371 (55.1)
Apgar score ≤4 after 5 min				
Apgar ≤4, *n* (%)	243 (0.6)	35 (1.0)	61 (0.4)	147 (0.7)
Perinatal death				
Perinatal death, *n* (%)	123 (0.3)	18 (0.5)	22 (0.1)	83 (0.4)

**Table 5:** Unadjusted and adjusted^*^ odds ratios (OR) for 5-minute Apgar score ≤4 and perinatal mortality (GA 34^+0^–36^+6^).

Characteristics	Apgar score ≤4 after 5 minutes			Mortality intrapartum and ≤7 dgn postpartum		
GA 34^+0^–36^+6^ *N* 41440	*N* (%)	Unadjusted OR	Adjusted OR	*N* (%)	Unadjusted OR	Adjusted OR
		(95% CI)	(95% CI)		(95% CI)	(95% CI)
Parturition	243 (0.59)			123 (0.30)		
Onset and delivery in primary care	35 (0.08)	1.54 (1.06–2.22)	1.75 (1.20–2.55)	18 (0.04)	1.40 (0.84–2.33)	1.61 (0.96–2.21)
Onset primary and delivery in secondary care	61 (0.15)	0.60 (0.44–0.81)	0.72 (0.53–0.98)	22 (0.05)	0.38 (0.24–0.61)	0.49 (0.30–0.79)
Onset and delivery in secondary care	147 (0.35)	Reference	Reference	83 (0.2)	Reference	Reference
Maternal age						
<25 years		0.98 (0.63–1.51)	0.92 (0.59–1.44)		1.11 (0.62–1.99)	1.14 (0.63–2.06)
25–29 years		Reference	Reference		Reference	Reference
30–34 years		1.24 (0.91–1.69)	1.9 (0.86–1.61)		1.18 (0.76–1.48)	1.05 (0.67–1.63)
≥35 years		1.37 (0.94–1.98)	1.13 (0.77–1.67)		1.27 (0.75–2.15)	0.88 (0.51–1.53)
Parity						
0		0.72 (0.54–0.96)	0.78 (0.58–1.05)		0.78 (0.51–1.20)	0.82 (0.53–1.27)
1		Reference	Reference		Reference	Reference
2+		1.39 (0.97–2.00)	1.30 (0.90–1.90)		2.13 (1.32–3.44)	2.14 (1.31–3.51)
Ethnicity						
European white		Reference	Reference		Reference	Reference
Non-European white		1.21 (0.88–1.68)	1.03 (0.73–1.47)		0.97 (0.60–1.59)	0.72 (0.43–1.21)
Socioeconomic status						
High		Reference	Reference		Reference	Reference
Medium		1.24 (0.88–1.75)	1.24 (0.88–1.75)		1.15 (0.72–1.85)	1.10 (0.68–1.77)
Low		1.47 (1.02–2.13)	1.39 (0.94–2.04)		1.40 (0.84–2.33)	1.22 (0.72–2.08)
Fetal sex						
Female		Reference	Reference		Reference	Reference
Male		0.91 (0.77–1.17)	0.89 (0.69–1.15)		0.89 (0.62–1.26)	0.86 (0.60–1.22)
Gestational age						
34^0^–34^6^		1.87 (1.35–2.57)	2.14 (1.55–2.96)		2.00 (1.27–3.17)	2.38 (1.491–3.78)
35^0^–35^6^		1.51 (1.13–2.02)	1.68 (1.25–2.25)		1.82 (1.21–2.73)	2.06 (1.37–3.11)
36^0^–36^6^		Reference	Reference		Reference	Reference

^*^Adjusted for onset and location of delivery, maternal age, parity before study delivery, ethnicity, socioeconomic status, fetal sex, and gestational age.
